# A Case Report of Deep Cerebral Venous Thrombosis Presenting Unilateral Lesion: The Association of Asymmetric Venous Outflow and Unilateral Lesion

**DOI:** 10.7759/cureus.36988

**Published:** 2023-04-01

**Authors:** Seiya Oshima, Hajime Ikenouchi, Tatsuo Miyamoto, Naoki Yamamoto, Kaoru Endo

**Affiliations:** 1 Neurology, Sendai City Hospital, Sendai, JPN

**Keywords:** anticoagulant therapy, asymmetric venous outflow, hypoplasia of the left transverse sinus, unilateral lesion, deep cerebral venous thrombosis

## Abstract

A 60-year-old man was admitted to our hospital due to progressive aphasia and right hemiparesis. Brain magnetic resonance imaging showed the left thalamus and basal ganglia lesion. Digital subtraction angiography showed the vein of Galen and straight sinus occlusion, suggesting cerebral venous thrombosis. Since his left transverse sinus was hypoplastic, his left deep cerebral lesion was due to the left deep cerebral vein congestion by the asymmetrical venous outflow. After anticoagulant therapy, his symptom and unilateral lesion improved. Clinicians should consider the vein of Galen and straight sinus thrombosis even in unilateral deep cerebral lesions.

## Introduction

Venous sinus thrombosis causes venous congestion and subsequently causes venous infarction or hemorrhage due to a thrombus in a cerebral vein, and its imaging findings are independent of the arterial vascular-dominant region [1]. The vein of Galen and straight sinus thrombosis sometimes presents with extensive venous infarction and can be life-threatening [2] and early diagnosis and treatment are essential. The vein of Galen and straight sinus thrombosis usually cause deep cerebral venous congestion such as the internal cerebral vein and the basal vein of Rosenthal, resulting in venous infarction or hemorrhage in bilateral deep cerebral lesion [3]. However, very rarely, the vein of Galen and straight sinus thrombosis present unilateral deep cerebral lesions [4,5]. Although unilateral deep cerebral lesions by straight sinus thrombosis have been reported to be more common on the left [6,7], their mechanism has not been fully investigated. We report a case of the vein of Galen and straight sinus thrombosis with unilateral deep cerebral lesions which would be derived from the anatomic asymmetrical deep cerebral venous outflow.

## Case presentation

**Figure 1:**
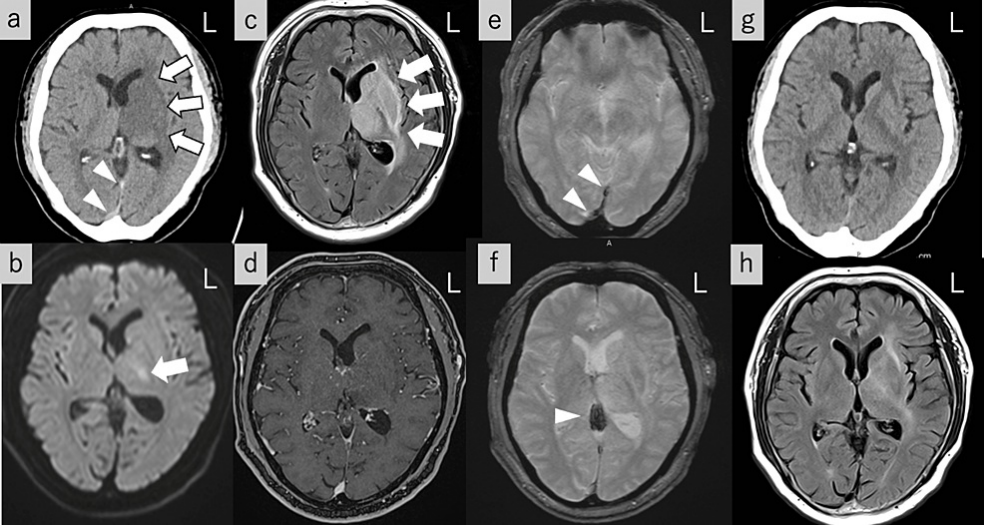
Diagnostic and follow-up findings of the present case. a: Axial computed tomography (CT) showed edematous changes in the left thalamus to basal ganglia (arrows) and high-density lesion in the straight sinus (arrowheads). b: Diffusion-weighted imaging showing high-intensity lesion confined to the basal ganglia (arrow). c: Fluid-attenuated inversion recovery (FLAIR) showed broad high-intensity lesions with edematous changes in the left thalamus and basal ganglia (arrows). d: Contrast-enhanced magnetic resonance imaging showed no brain tumor in the left thalamus and basal ganglia. e,f: T2*-weighted imaging (T2*WI) showed low intensity suggesting thrombus in the vein of Galen to straight sinus (arrowheads). g: Follow-up CT showed improvement of edematous changes in the left thalamus to basal ganglia. h: Follow-up FLAIR showed improvement of the left thalamus and basal ganglia lesion.

A 60-year-old man came to our emergency room due to progressive aphasia for two days. He had a history of hypertension and dyslipidemia. He had no family history including hematological and immunological diseases. He also had no recent vaccination history, and no medications that could cause thrombosis. On arrival at the emergency room, he had mild consciousness disturbance with Glasgow Coma Scale (GCS) of 14 (E4V4M6) and slight motor aphasia. He had no hemiparesis or sensory disturbances. However, the right hemiparesis gradually emerged within an hour after arrival at the emergency room and motor aphasia also deteriorated. Brain computed tomography (CT) showed broad edematous changes in the left thalamus and basal ganglia (Figure 1A). CT also showed a high-density lesion in the straight sinus (Figure 1A), suspected straight sinus thrombosis. In brain magnetic resonance imaging (MRI), diffusion-weighted images (DWI) showed a high-intensity lesion in the left thalamus (Figure 1B) with an apparent diffusion coefficient decrement. Fluid attenuated inversion recovery (FLAIR) showed broad lesions with edematous change in the left thalamus and basal ganglia (Figure 1C). Cerebral infarction, venous sinus thrombosis, and brain tumor were differential diagnoses. As a broad unilateral lesion in the thalamus and basal ganglia would be atypical for cerebral infarction and venous sinus thrombosis, we first suspected a brain tumor. However, contrast-enhanced MRI showed no suggestive findings of brain tumor (Figure 1D). T2*-weighted image (T2*WI) showed low intensity in the vein of Galen to straight sinus (Figure 1E, 1F). Based on CT and MRI findings, we diagnosed cerebral venous sinus thrombosis. 

**Figure 2:**
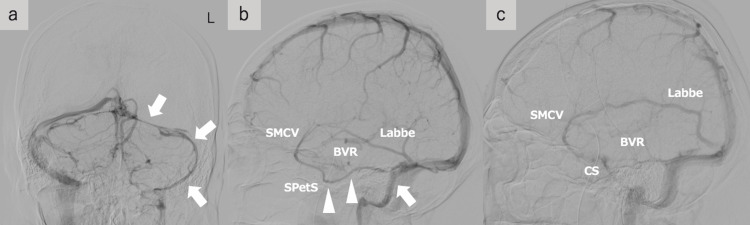
Digital subtraction angiography findings of the present case a: Vertebral artery angiography showed the left transverse and sigmoid sinus were hypoplastic (arrows). b,c: Bilateral internal carotid artery (ICA) angiography (b: left ICA and c: right ICA) showed the drainage route of the internal cerebral vein – the vein of Galen – straight sinus was not delineated (b and c), and the left basal vein of Rosenthal (BVR) was higher congested than the right (b). The left superficial middle cerebral vein (SMCV) was refluxing into the hypoplastic left transverse sinus (arrow) as sphenopetrosal sinus (SPetS) without going through the cavernous sinus (CS) (b) (arrowheads).

In the vertebral artery digital subtraction angiography), the left transverse sinus and sigmoid sinus were hypoplastic (Figure 2A). In the bilateral internal carotid artery (ICA) digital subtraction angiography (DSA), the bilateral internal cerebral vein, the vein of Galen, and straight sinus were occluded, and the left basal vein of Rosenthal (BVR) showed higher congestion than the right (Figure 2B, 2C). There was no obvious anomalous drainage except for the left superficial middle cerebral vein (SMCV) refluxing into the hypoplastic left transverse sinus and sigmoid sinus through the left sphenopetrosal sinus (SPetS) (Figure 2B).

The diagnosis of the vein of Galen and straight sinus thrombosis was made. His left deep cerebral lesion would be due to the left deep cerebral vein congestion by the asymmetrical venous outflow based on the left hypoplastic transverse sinus and anomalous drainage route. Since his symptom was progressive, we started heparin immediately after the diagnosis of cerebral venous thrombosis. His symptom progression stopped after heparin and gradually improved. Therefore, we did not conduct angioplasties for occluded venous sinus. Follow-up CT and FLAIR on day nine after admission showed remission of left basal ganglia and thalamic lesion (Figure 1G, 1H). Since blood tests showed no abnormalities of thrombogenic predisposition such as infection, disseminated intravascular coagulation, protein C and S abnormalities, anti-phospholipid antibodies, and autoantibodies (Table 1), the cause of venous sinus thrombosis was unknown.

**Table 1:** Laboratory findings of the present case Laboratory tests showed no elevated inflammatory response , not prolonged prothrombin time-international normalized ratio, slightly elevated D-dimer level, elevated γ-GTP level, no electrolyte imbalance. The additional blood test for autoimmune antibodies such as anti-nuclear antibodies, antiphospholipid antibodies and protein C and S autoantibodies were negative. Abbreviations: WBC, white blood cell; Hb, hemoglobin; Plt, platelets; PT-INR, prothrombin time-international normalized ratio; APTT, activated partial thromboplastin time; AST, aspartate aminotransferase; ALT, alanine aminotransferase; γGTP; γ-glutamyl trans peptidase; BUN, blood urea nitrogen; CRP, C-reactive protein; ANA, anti-nuclear antibody; APA, antiphospholipid antibody; ATⅢ, antithrombin Ⅲ.

Test	Value	Normal range	Test	Value	Normal range
WBC	7300 /μL	3100-8400	BUN	14 mg/dL	8-20
Hb	15.1 g/dL	11.4-14.6	Creatinine	0.79 mg/dL	0.46-0.79
Plt	19.9×10^3^ /μL	15-40	Na	142 mmol/L	135-145
PT-INR	0.97	0.85-1.15	K	3.9 mmol/L	3.5-4.5
APTT	23.7 sec	25-40	Cl	106 mmol/L	97-106
D-dimer	1.05 μg/mL	<1.0	CRP	0.20 mg/dL	<0.3
AST	28 U/L	13-33	ANA	(-)	
ALT	41 U/L	8-42	APA	(-)	
γ-GTP	85 U/L	<65	Protein C	122 %	70-150
Total protein	6.4 g/dL	6.5-8.0	Protein S	129	67-164
Albumin	3.7 g/dL	3.5-5.0	ATⅢ	103%	80-130

**Figure 3:**
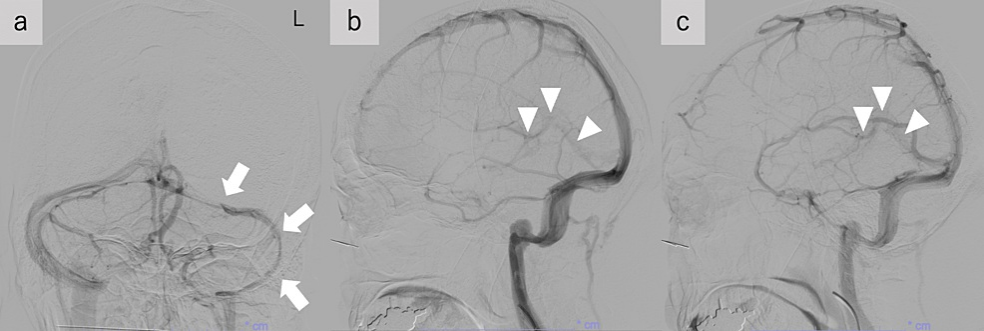
Follow-up digital subtraction angiography findings of the present case a: Vertebral artery angiography showed the left transverse and sigmoid sinus were hypoplastic (arrows). b,c: Bilateral internal carotid artery (ICA) angiography (b: left ICA and c: right ICA) showed no occlusion in the internal cerebral vein – the vein of Galen – straight sinus (arrowheads) (b and c).

We used heparin for the treatment of cerebral venous thrombosis and switched to direct oral anticoagulants [8] on day 11. His right hemiparesis completely improved, but slight aphasia remained. He was transferred to a rehabilitation hospital on day 17. Follow-up DSA at nine months after onset complete remission of Galen to Straight sinus thrombosis (Figure 3A-3C).

## Discussion

This is a case of the vein of Galen and straight sinus thrombosis with a unilateral deep cerebral lesion. Although suspected findings of cerebral venous thrombosis were present at first brain CT, we first suspected brain tumor because of the unilateral lesion. However, contrast-enhanced MRI was normal, and T2*WI showed an obvious thrombus in the venous sinus, which led to the diagnosis of deep cerebral venous thrombosis. Despite the progressive symptoms, early diagnosis of cerebral venous thrombosis and treatment by anticoagulant therapy would succeed in the symptom and lesion remission.

The vein of Galen and straight sinus thrombosis typically show bilateral thalamic lesions [3]. However, very rarely, the vein of Galen and straight sinus thrombosis shows a unilateral deep cerebral lesion, especially on the left side [6,7]. Although the asymmetry of the venous outflow tract may be speculated as one possible mechanism [7], imaging has not confirmed its mechanism. In the present case, the left BVR showed higher congestion than the right. The asymmetry of BVR congestion would suggest the asymmetrical drainage route. Another venous drainage was almost normal, except for the hypoplastic left transverse sinus and the left SMCV refluxing into the left hypoplastic transverse and sigmoid sinus through SPetS. therefore, the higher congestion in the left BVR than in the right suggests that a higher proportion of the left deep venous drainage is dependent on the vein of Galen to straight sinus outflow than on the right. This could suggest that the vein of Galen and straight sinus occlusion would easily cause venous infarction in the left hemisphere than the right. The transverse sinus is reported to be more hypoplastic on the left [9]. Therefore, the previous cases of the unilateral lesion would be due to the asymmetrical venous outflow as shown in the present case. The vein of Galen and straight sinus thrombosis sometimes presents with extensive venous infarction and can be life-threatening [2] and early diagnosis and treatment are essential. In the present case, although symptoms showed progression, early diagnosis and treatment would improve symptoms and brain lesions. Since anatomical asymmetry of the venous outflow may contribute to the unilateral deep cerebral lesion, clinicians should be aware of these rare imaging characteristics and consider the vein of Galen and straight sinus thrombosis even in unilateral deep cerebral lesions.

## Conclusions

The vein of Galen and straight sinus thrombosis sometimes present with extensive venous infarction and can be life-threatening. Even in the case of unilateral deep cerebral lesions, cerebral venous thrombosis should be considered a differential diagnosis. Anatomical asymmetry of the venous outflow may contribute to the unilateral deep cerebral lesion.
